# Contributions of Hippocampal Volume to Cognition in Healthy Older Adults

**DOI:** 10.3389/fnagi.2020.593833

**Published:** 2020-11-05

**Authors:** Cheshire Hardcastle, Andrew O’Shea, Jessica N. Kraft, Alejandro Albizu, Nicole D. Evangelista, Hanna K. Hausman, Emanuel M. Boutzoukas, Emily J. Van Etten, Pradyumna K. Bharadwaj, Hyun Song, Samantha G. Smith, Eric C. Porges, Steven Dekosky, Georg A. Hishaw, Samuel S. Wu, Michael Marsiske, Ronald Cohen, Gene E. Alexander, Adam J. Woods

**Affiliations:** ^1^Center for Cognitive Aging and Memory, College of Public Health and Health Professions, McKnight Brain Institute, University of Florida, Gainesville, FL, United States; ^2^Department of Clinical and Health Psychology, College of Public Health and Health Professions, University of Florida, Gainesville, FL, United States; ^3^Department of Neuroscience, College of Medicine, University of Florida, Gainesville, FL, United States; ^4^Evelyn F. McKnight Brain Institute, University of Arizona, Tucson, AZ, United States; ^5^Department of Psychology, School of Mind, Brain and Behavior, College of Science, University of Arizona, Tucson, AZ, United States; ^6^Department of Neurology, College of Medicine, University of Florida, Gainesville, FL, United States; ^7^Department of Neurology, University of Arizona, Tucson, AZ, United States; ^8^Department of Psychiatry, University of Arizona, Tucson, AZ, United States; ^9^Department of Biostatistics, University of Florida, Gainesville, FL, United States; ^10^Neuroscience Graduate Interdisciplinary Program, University of Arizona, Tucson, AZ, United States; ^11^Physiological Sciences Graduate Interdisciplinary Program, University of Arizona, Tucson, AZ, United States; ^12^Arizona Alzheimer’s Consortium (AAC), Phoenix, AZ, United States

**Keywords:** aging, cognition, hippocampus, magnetic resonance imaging, brain volume, NIH toolbox

## Abstract

**Objective**: The association between hippocampal volume and memory is continuing to be characterized in healthy older adults. Prior research suggests smaller hippocampal volume in healthy older adults is associated with poorer episodic memory and processing speed, as well as working memory, verbal learning, and executive functioning as measured by the NIH Toolbox Fluid (Fluid Cognition Composite, FCC) and Crystalized Cognition Composites (CCC). This study aimed to replicate these findings and to evaluate the association between: (1) hippocampal asymmetry index and cognition; and (2) independent contributions of the left and right hippocampal volume and cognition in a large sample of healthy older adults.

**Participants and Methods**: One-hundred and eighty-three healthy older adults (M age = 71.72, SD = 5.3) received a T1-weighted sequence on a 3T scanner. Hippocampal subfields were extracted using FreeSurfer 6.0 and combined to provide left, right, and total hippocampal volumes. FCC subtests include Dimensional Change Card Sort, Flanker Inhibitory Control and Attention, List Sorting, Picture Sequence Memory, and Pattern Comparison. CCC subtests include Picture Vocabulary and Oral Reading Recognition. Multiple linear regressions were performed predicting cognition composites from the total, left and right, and asymmetry of hippocampal volume, controlling for sex, education, scanner, and total intracranial volume. Multiple comparisons in primary analyses were corrected using a false discovery rate (FDR) of *p* < 0.05.

**Results**: FCC scores were positively associated with total (*β* = 0.226, FDR *q* = 0.044) and left (*β* = 0.257, FDR *q* = 0.024) hippocampal volume. Within FCC, Picture Sequence Memory scores positively associated with total (*β* = 0.284, *p* = 0.001) and left (*β* = 0.98, *p* = 0.001) hippocampal volume. List Sorting scores were also positively associated with left hippocampal volume (*β* = 0.189, *p* = 0.029).

**Conclusions**: These results confirm previous research suggesting that bilateral hippocampal volume is associated with FCC, namely episodic memory. The present study also suggests the left hippocampal volume may be more broadly associated with both episodic and working memory. Studies should continue to investigate lateralized hippocampal contributions to aging processes to better identify predictors of cognitive decline.

## Introduction

The hippocampus is a bilateral medial temporal lobe structure known for its important role in episodic learning and memory function, or the ability to remember ongoing experiences (Vargha-Khadem et al., 1997; Tulving and Markowitsch, 1998). Hippocampal volume has been associated with domains of cognition such as processing speed, working memory, spatial navigation, and abstract reasoning (Reuben et al., 2011; Lövdén et al., 2012; O’Shea et al., 2016; Gorbach et al., 2017). These cognitive domains are particularly vulnerable to a decline in non-pathological aging (Salthouse, 2009), and therefore have been further studied in the context of hippocampal volume reduction in cognitive aging.

O’Shea et al. (2016) examined the relationship between cognitive functioning and hippocampal volume using NIH Toolbox’s fluid and crystalized cognition composite (CCC) scores in a sample of 93 older adults. The authors found smaller bilateral hippocampal volume was associated with poorer performance on fluid cognitive functioning. The greatest magnitude of association was with episodic memory and processing speed, but also included working memory, verbal learning, and executive functions. Findings from this study were consistent with prior research supporting a clear role of the hippocampus in episodic memory and also suggest hippocampal volume may play a broader role in cognitive aging other than memory, such as processing speed.

While O’Shea et al. (2016) assessed combined bilateral hippocampal volume, previous studies have demonstrated asymmetry in hippocampal volume in healthy older adults, with the right volume typically larger than left (Woolard and Heckers, 2012; Hou et al., 2013). Additionally, there are differences in hippocampal volume asymmetry across dementia disease progression, notably in mild cognitive impairment (MCI). Through a meta-analysis, Shi et al. (2009) evaluated the asymmetry of the hippocampus in healthy older adults, older individuals with MCI, and those with Alzheimer’s disease (AD). Their findings revealed an overall pattern of right greater than left asymmetry in all groups. However, adults with MCI had the most pronounced right greater than left asymmetry when compared to healthy adults and those with AD (Shi et al., 2009).

In addition to volume asymmetry, lateralized cognitive functioning as it relates to hippocampal volume has been explored in pathological populations. Delaney et al. (1980) compared epileptic patients with seizure foci originating in either the left or right temporal lobe on verbal and non-verbal memory tasks. Their findings revealed a dissociation between verbal and non-verbal memory deficits, such that patients with right temporal lobe epilepsy were more impaired on non-verbal memory tasks, while patients with left temporal lobe epilepsy were more impaired on verbal memory tasks. In a small sample (*n* = 36) of healthy older adults and adults with MCI, Müller et al. (2005) found a relationship between left hippocampal volume only and a verbal memory task in the MCI group. These findings suggest cognitive function laterality associated with hippocampal volume in non-healthy patients, although this same relationship has yet to be studied in large samples of healthy older adults.

In summary, the relationship between bilateral hippocampal volume and performance in specific cognitive domains (i.e., episodic memory, verbal learning, processing speed, and executive function) has been characterized by only a few studies in healthy older adults (Woolard and Heckers, 2012; O’Shea et al., 2016). However, previous research shows hemispheric differences in hippocampal functioning, and asymmetry in hippocampal volume decline in the dementia process (Delaney et al., 1980; Müller et al., 2005; Shi et al., 2009). There is currently a gap in the literature characterizing the relationship between asymmetry/lateral hippocampal volume and cognitive functioning in healthy older adults. We must first understand cognitive differences in lateral hippocampal volume in healthy aging to further understanding of pathological aging.

The present study aims to fill this gap by first replicating findings from O’Shea et al. (2016) with a larger independent sample, and then by assessing the relationship of asymmetry and laterality of hippocampal volume and cognitive functioning in healthy older adults. We hypothesized that there would be a positive association between bilateral hippocampal volume and fluid cognitive functioning, namely episodic memory and processing speed. Given a lateralized relationship between cognitive functioning and hippocampal volume in pathological adults, we predict that there will also be a similar relationship in healthy older adults. Specifically, we predict that verbally loaded memory tasks may have a more robust relationship with left hippocampal volume.

## Materials and Methods

### Participants

One hundred and eighty-three older adults (mean age = 71.72; SD = 5.3) were drawn from two ongoing randomized clinical trials with identical inclusion/exclusion criteria (R01AG054077; K01AG050707) across the University of Florida (UF; *n* = 126) and the University of Arizona (AU; *n* = 57; Woods et al., 2018). Participant demographic characteristics are shown in Table 1. Only baseline data were included in this study. The participants were recruited through newspaper advertising, flyers, and community outreach. All participants provided written informed consent before study procedures began, and all procedures were approved by the Institutional Review Boards at the University of Florida and the University of Arizona. Participants were excluded for left-handedness, if they were outside the age range of 65–89, had a history of brain or head injury that resulted in a loss of consciousness for greater than 20 min, identified a pre-existing neurological condition, psychiatric disorder, MRI contraindications (e.g., medical devices or implants not approved for 3T MRI), diagnosis of neurodegenerative brain disease (i.e., AD, Parkinson’s disease, amyotrophic lateral sclerosis), or self-reported difficulties in thinking and/or memory. At the in-person screening visit, participants were further screened for MCI through the use of the Unified Data Set (UDS) of the National Alzheimer’s Coordinating Center (NACC; Weintraub et al., 2009) and administration of the Montreal Cognitive Assessment (MoCA). If they were equal to or below 1.5 standard deviations on any cognitive domain including general cognition, visuospatial functioning, executive functioning/working memory, or language, they were not included. For more detail on inclusion/exclusion criteria, refer Woods et al. (2018).

**Table 1:** Sample demographics.

	University of Florida (*n* = 126)	University of Arizona (*n* = 57)	Combined (*n* = 183)
Age M (SD), range	71.85 (5.8), 65–88	71.44 (4.1), 65–80	71.72 (5.3), 65–88
Education M (SD), range	16.09 (2.5), 12–21	16.12 (2.0), 12–20	16.10 (2.3), 12–21
Sex (Ma:F)	54:72	18:39	72:111
MoCA M (SD), range	26.70 (1.9), 21–30	27.00 (1.9), 22–30	26.80 (1.9), 21–30

*Note. M, mean; SD, standard deviation; Ma, male; F, female; MoCA, Montreal Cognitive Assessment*.

### Study Procedures

At the study baseline visit, all participants completed a neuropsychological battery that included the NIH Toolbox—Cognition Battery (NIHTB-CB). All neuropsychological tasks were administered at an onsite clinical research facility by trained study staff. The NIHTB-CB battery and MRI scan were completed on the same day.

### NIH Toolbox Cognition Battery

The NIH Toolbox Cognition Battery is a brief yet valid and comprehensive assessment of cognitive functioning in a variety of domains (Heaton et al., 2014). For this study, we utilized the unadjusted standard scores of the Fluid Cognition Composite (FCC) and the Crystalized Cognitive Composite (CCC). FCC is comprised of cognitive domains that have been shown to decline with age, while CCC is comprised of language-based assessments that are stable or even improve with age (Horn and Cattell, 1967).

The FCC is comprised of five domains of cognitive functioning. The domains and description of NIHTB-CB subtask used to assess these domains are as follows:

Processing Speed—Pattern Comparison Task asks participants to rapidly match visual patterns.
Attention/Inhibition—Flanker Inhibitory Control and Attention task assess visual attention and inhibition by asking participants to determine the direction of a target arrow that is flanked by stimuli on left and right.
Executive Functioning—Dimensional Card Sort task assesses set-shifting by asking participants to match cards based on switching between multiple rules and strategies.
Working Memory—List Sorting task asks participants to sort lists of visual and auditory stimuli from smallest to largest.
Episodic Memory—Picture Sequence Memory task asks participants to remember a sequence of thematically related pictures.

The CCC is comprised of two domains assessing language functioning. The description of NIHTB-CB subtask used to assess this domain is as follows:

Oral Reading Recognition Task—The participant is asked to read and pronounce words as accurately as possible.
Picture Vocabulary Test—The participant is asked to match auditorily presented words to pictures.

### Image Acquisition

All participants received a high-resolution 3D T1-weighted MPRAGE sequence on a 3-Tesla Siemens Magnetom Prisma Scanner with a 64-channel head coil at the Center for Cognitive Aging and Memory at the University of Florida and a 3-Tesla Siemens Magnetom Skyra scanner with a 32-channel head coil at the University of Arizona. Both study sites followed identical sequence and scanning procedures, including reducing head motion through the use of foam padding, and the use of earplugs to reduce scanner noise. Scanning parameters are as follows: echo time (TE) = 2.26 ms; repetition time (TR) = 1,800 ms; flip angle = 8°; FOV = 256 × 256 × 176 mm, voxel size = 1 mm^3^.

### Image Processing

T1-weighted images were processed with the FreeSurfer software version 6.0, freely available to download at http://surfer.nmr.mgh.harvard.edu/. All images were processed using the “recon-all” pipeline, which briefly includes motion correction and averaging (Reuter et al., 2010), skull stripping (Ségonne et al., 2004), Talaraich transformation, intensity normalization (Sled et al., 1998), gray/white matter segmentation, and topology correction (Fischl et al., 2001; Ségonne et al., 2007). A more detailed description of this process can be found at Fischl et al. (2001) and Ségonne et al. (2004).

Within FreeSurfer 6.0, the hippocampal segmentation module was applied to extract 12 subfields of bilateral hippocampi that were summed to produce total hippocampal volume (Iglesias et al., 2015, 2016; Saygin et al., 2017). This tool uses Bayesian inference from a probabilistic atlas built with ultra-high resolution *ex vivo* MRI data (~0.1 mm isotropic)^1^. While this tool segments hippocampal and amygdala subfields, only total left and right hippocampal volumes derived from subfield segmentation were used in this study. This tool has been validated against manual segmentation in older adult brains and was shown to have higher accuracy in segmentation due to the use of *ex vivo* segmentation techniques that better match with histological studies (Iglesias et al., 2015; Schmidt et al., 2018). Additionally, this atlas is better able to discriminate between AD and control participants using whole hippocampal volume, suggesting total hippocampal measurement may be more accurate than the previous *in vivo* mask (Iglesias et al., 2015). While hippocampal subfields have been shown to differentially decline with age (Zheng et al., 2018), we were interested in replicating previous findings with a larger independent sample (O’Shea et al., 2016) and exploring differences in left-right total hippocampal volume and cognitive functioning, and thus chose to only assess whole hippocampal volume.

**Figure 1:**
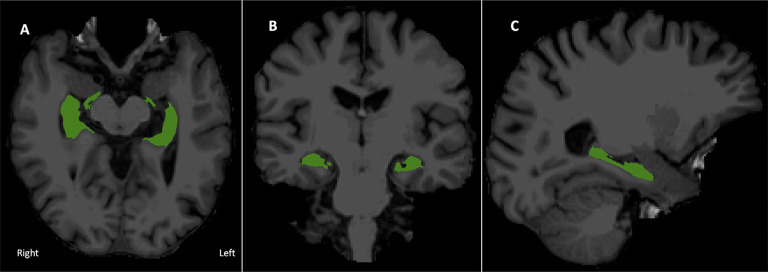
Bilateral hippocampal region of interest overlaid onto the T1 image. **(A)** Axial, **(B)** coronal, and **(C)** right sagittal view of combined hippocampal subfields in radiological display convention.

See Figure 1 for a visual depiction of hippocampal volume.

### Statistical Analysis

All statistical analyses were performed in SPSS software version 25. UF and UA study sites were compared on demographic factors, then combined. Outlier analyses were conducted on dependent variables. Any participants who had cognitive *z*-scores in any subtest greater than three standard deviations away from the mean were excluded in further analyses. NIHTB-CB descriptive statistics can be found in Table 2.

**Table 2:** NIH Toolbox-Cognition Battery (NIHTB-CB) fluid cognition and crystalized cognition composites and subtests.

	Mean (*n* = 183)	SD	Range
CCC	116.03	7.03	92–132
Pic. Vocab	117.90	8.68	93–139
Oral Recog.	112.66	5.59	93–125
FCC	92.54	8.86	62–112
Pattern Comp.	89.64	14.46	55–122
Flanker	93.64	6.77	66–106
Card Sort	100.60	7.32	77–120
List Sorting	98.27	9.10	74–124
Pic. Seq.	95.31	10.34	76–126

*Note. SD, standard deviation; CCC, Crystalized Cognition Composite; FCC, Fluid Cognition Composite. All scores are in a standard score format (mean = 100, SD = 10)*.

To measure asymmetry, an asymmetry index was calculated as the ratio of the absolute difference of left from right hippocampal volume to the sum of left and right hippocampal volume (Sarica et al., 2018). Therefore, values closer to zero represent no asymmetry. The absolute difference was calculated to represent the magnitude of asymmetry that does not indicate laterality.

$$\text{Asymmetry Index}=\frac{\left|\text{L}-\text{R}\right|}{\text{L}+\text{R}}$$

Primary analysis assessed total, asymmetry index, and lateralized contribution of hippocampal volume to NIHTB-CB cognitive composites. Linear regressions were performed predicting FCC and CCC from combined, asymmetry ratio, then left and right hippocampal volume separately, totaling to eight separate regression models. Sex, education, scanner type, and total intracranial volume were entered into all models as covariates. Multiple comparisons in primary analysis were corrected using a false discovery rate (FDR) of *p* < 0.05. Significant primary analyses were followed up by secondary linear regressions to assess NIHTB-CB subtest performance with hippocampal volume variables.

## Results

### Replication of Total Hippocampal Volume and NIHTB-CB

**Figure 2:**
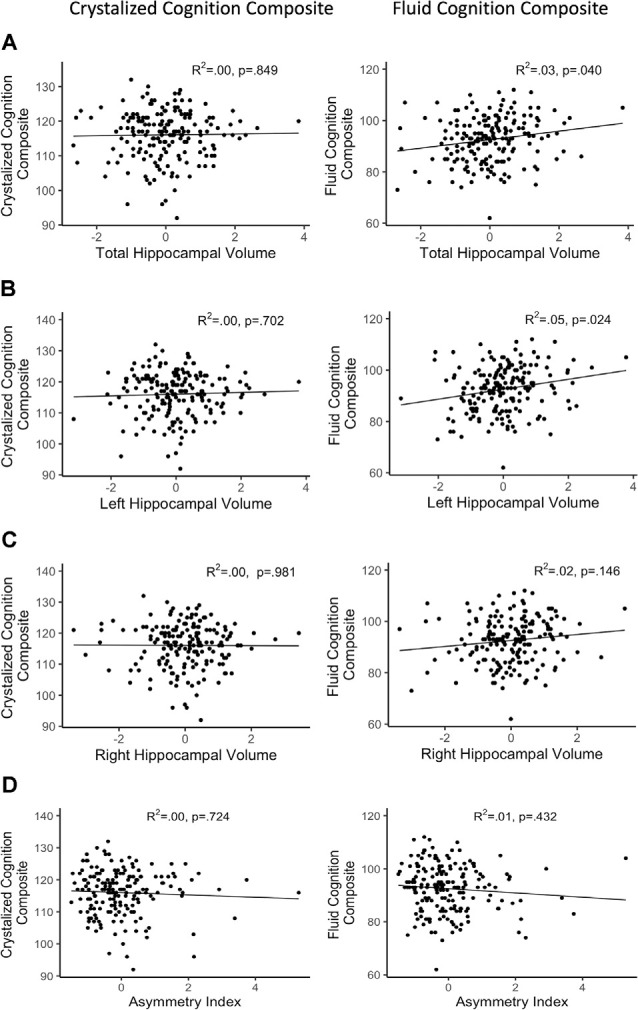
Regressions of hippocampal volume and NIHTB cognition composites. **(A)** Total hippocampal volume; **(B)** left hippocampal volume; **(C)** right hippocampal volume; **(D)** asymmetry index; R^2^ reflects variance explained from the partial correlation between hippocampal volume and NIHTB-CB score; X-axis = residual of hippocampal volume after controlling for sex, education, scanner, and total intracranial volume; Y-axis = standard score of NIHTB-CB variables.

**Figure 3:**
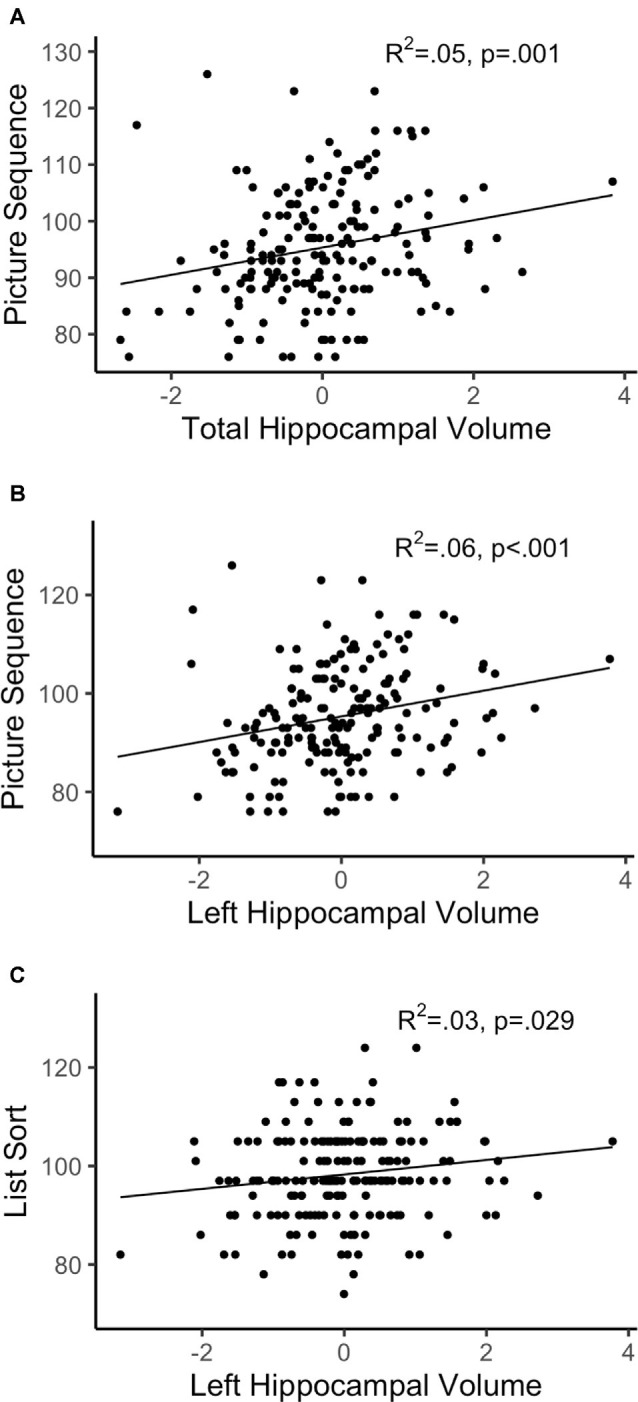
Regressions of hippocampal volume and significant subtests. **(A)** Total hippocampal volume; **(B,C)** left hippocampal volume; R^2^ reflects variance explained from the partial correlation between hippocampal volume and NIHTB-CB score; X-axis = residual of hippocampal volume after controlling for sex, education, scanner, and total intracranial volume; Y-axis = standard score of Fluid Cognition Composite (FCC) subtest variables.

After FDR adjustment of the *p*-value (*q*-value), total hippocampal volume significantly positively predicted the FCC (*q* < 0.05) but was not related to the CCC. Therefore, we did not explore the relationship between subtest performance within CCC and total hippocampal volume. Out of the subtests within FCC, total hippocampal volume was significantly positively associated with the episodic memory subtest, picture sequence memory (*p* < 0.001). Total hippocampal volume did not significantly predict any other subtest within FCC. Refer Table 3 for complete statistical output and Figures 2A, 3A for scatter plot visual representation.

**Table 3:** Regressions of total hippocampal volume and NIH Toolbox—Cognition Battery (NIHTB-CB).

	**β**	*t* (df = 177)	*p/q*-value
CCC	0.027	0.328	0.849
FCC	0.226	2.579	0.044*
Pattern Comp.	0.121	1.332	0.184
Flanker	0.114	1.293	0.198
Card Sort	0.055	0.619	0.536
List Sorting	0.153	1.737	0.084
Pic. Seq.	0.284	3.360	0.001***

Note. CCC, Crystalized Cognition Composite; FCC, Fluid Cognition Composite; β, Standardized Beta; CCC and FCC *p*-values are FDR adjusted; *p < 0.05; ***p < 0.001.

### Asymmetry of Hippocampal Volume and NIHTB-CB

After FDR adjustment, the asymmetry index of the hippocampus did not significantly predict FCC or CCC. Therefore, the relationship of subtest scores and asymmetry of the hippocampal volume was not explored for either cognition composite. Refer Table 4 for complete statistical output and Figure 2D for scatter plot visual representation.

**Table 4:** Regressions of asymmetry index and NIHTB-CB.

	*β*	*t* (df = 177)	*q*-value
CCC	−0.053	−0.752	0.724
FCC	−0.094	−1.242	0.432

*Note. CCC, Crystalized Cognition Composite; FCC, Fluid Cognition Composite; CCC and FCC *p*-values are FDR adjusted; β, Standardized Beta*.

### Left and Right Hippocampal Volume and NIHTB-CB

After FDR adjustment, left hippocampal volume significantly positively predicted FCC (*q* < 0.05), but not CCC. The right hippocampal volume did not significantly predict the FCC or CCC. Therefore, the relationship between subtest performance and hippocampal volume was only explored with left hippocampal volume for the FCC subtests. Out of the FCC subtests, left hippocampal volume was significantly positively associated with the working memory subtest, list sorting (*p* < 0.05), and the episodic memory subtest, picture sequence memory (*p* < 0.001). Left hippocampal volume did not significantly predict any other subtest within FCC. Refer Table 5 for complete statistical output and Figure 2B (left) and Figure 2C (right) for NIHTB-CB composite scatter plot visual representation, and Figures 3B,C for NIHTB FCC subtest scatter plot visual representation.

**Table 5:** Regressions of lateral hippocampal volume and NIHTB-CB.

	Left hippocampus			Right hippocampus		
	*β*	*t* (df = 177)	*p/q*-value	*β*	*t* (df = 177)	*q*-value
CCC	0.052	0.634	0.702	−0.002	−0.023	0.981
FCC	0.257	3.033	0.024*	0.158	1.801	0.194
Pattern Comp.	0.126	1.424	0.156			
Flanker	0.168	1.986	0.051			
Card Sort	0.07	0.809	0.420			
List Sorting	0.189	2.207	0.029*			
Pic. Seq.	0.298	3.643	<0.001***			

Note. CCC, Crystalized Cognition Composite; FCC, Fluid Cognition Composite; β, Standardized Beta. CCC and FCC *p*-values are FDR adjusted; *p < 0.05; ***p < 0.001.

## Discussion

Smaller hippocampal volume in older adults is related to poorer cognitive functioning in domains of episodic memory, verbal learning, processing speed, and executive function (Woolard and Heckers, 2012; O’Shea et al., 2016). Additionally, previous research suggests that cognitive functions associated with the hippocampus may be lateralized, and this cognitive asymmetry may change with the aging process and progression into dementia (Delaney et al., 1980; Müller et al., 2005; Shi et al., 2009). This study sought to replicate and extend O’Shea et al.’s (2016) findings with a larger, and independent sample, confirming that greater hippocampal volume is associated with better fluid cognitive functioning as measured by the NIHTB-CB in older adults. We further sought to characterize the relationship between lateral and asymmetrical hippocampal volume and cognitive functioning in older adults.

### Total Hippocampal Volume and NIHTB-CB

Greater total hippocampal volume was related to higher scores on NIHTB FCC, after we controlled for sex, education, and total intracranial volume. Further, the episodic memory subtest (picture sequence memory) within the FCC showed the strongest relationship with total hippocampal volume. This replicates findings from O’Shea et al. (2016), who also found a positive relationship between total hippocampal volume and episodic memory. The replication of this relationship confirms the association between total hippocampal volume and episodic memory performance in aging. Prior data in patient populations with hippocampal sclerosis and hippocampal degeneration have also demonstrated the role of hippocampal volume in episodic memory (Sexton et al., 2010; Saghafi et al., 2018). However, our larger sample did not show a relationship between total hippocampal volume and processing speed, which O’Shea et al. (2016) found to be at near the same magnitude as episodic memory. Possibly this relationship was not robust enough to withstand a larger sample, and a possibly more accurate methodology of volume extraction (Iglesias et al., 2015).

### Asymmetry of Hippocampal Volume and NIHTB-CB

There is a reliable and reproducible finding of right-greater-than-left asymmetry of the hippocampus in healthy older adults (Shi et al., 2009; Sarica et al., 2018). *Post hoc* analyses confirmed this in our sample when comparing lateralized volume using a paired samples *t*-test (*t*_(182)_ = 6.248, *p* < 0.001). However, our findings show that this volumetric asymmetry is not related to fluid cognitive functioning in healthy older adults. Through a meta-analytical review of literature, Shi et al. (2009) were able to show that this asymmetry was greatest in the MCI portion of a trajectory to Alzheimer’s dementia, although other studies have shown greater asymmetry in a population with dementia due to AD (Sarica et al., 2018). A cognitive relationship with structural asymmetry may not yet be evident in healthy older adults. Similar results have been found in a study assessing the asymmetry of hippocampal volume and its relationship to learning and memory (Sarica et al., 2018). Sarica et al. (2018) focused on measures of learning and memory extracted from the Rey Auditory Verbal Learning Test (RAVLT). Nevertheless, they also did not find an association between an asymmetry index and cognitive functioning in healthy older adults. Interestingly, they were able to show this association only in a population with dementia due to AD. Taken together, our findings support previous literature suggesting that structural asymmetry of the hippocampus, while evident, is not necessarily related to differences in cognitive functioning assessed over a range of cognitive domains in cognitively healthy adults.

### Lateralization of Hippocampal Volume and NIHTB-CB

Despite no evidence of a relationship between structural asymmetry and cognition in this sample, our findings did suggest cognitive laterality. Left hippocampal volume had a positive association with fluid cognition scores, specifically the episodic and working memory subtests of the NIHTB FCC. Interestingly, right hippocampal volume showed no association with either fluid or crystallized cognition composites from the NIHTB. Left hippocampal volume is thought to relate to verbally-loaded memory tasks, while right hippocampal volume is thought to be related to spatially-loaded memory tasks (Delaney et al., 1980; Bonner-Jackson et al., 2015; Ezzati et al., 2016). Given our measure of episodic memory has a prominent verbal component, we predicted that left hippocampal volume would most strongly predict episodic memory. These results confirm previous research showing a positive association between verbally-based immediate and delayed measures of episodic memory and left hippocampal volume in healthy older adults (Ezzati et al., 2016).

Our findings also showed a positive relationship between working memory and left hippocampal volume. While the key neural correlates of working memory in aging are traditionally thought to include the function of the bilateral dorsolateral prefrontal cortex, inferior parietal lobes, and insula (Suzuki et al., 2018), other studies have suggested that the hippocampus may also play a role in this memory system. Through the use of combined functional MRI and intracranial electroencephalogram (EEG) recordings in epilepsy patients and healthy controls, Axmacher et al. (2007) demonstrated sustained activity in medial temporal lobes during visual working memory maintenance, and the amount of activation was related to working memory load. Some have argued that hippocampal involvement in maintenance is related to the process of long-term memory because maintenance predicts the number of items consolidated (Schon et al., 2004; Axmacher et al., 2007; Leszczynski, 2011). However, our working memory measure was derived from the sum of total items correctly sorted, rather than maintenance. Therefore, our findings expand upon this notion by suggesting that in cognitive aging left hippocampal volume may also be associated with aspects of working memory that is independent of long-term consolidation. The NIHTB-CB FCC working memory subtest also requires sorting of information presented in a visual and auditory format, rather than predominately verbal (Tulsky et al., 2014). Given this measure of working memory only associated with the left hippocampal volume, the left hippocampus may play a broader role in working memory functioning that isn’t limited to verbally presented information.

No cognition composite was significantly related to the right hippocampal volume. That is not to say that the right hippocampal volume does not play a role in cognition. Notably, FCC does explain ~2% of the variance in the right hippocampal volume. With increased power and broader cognitive assessments, possibly a significant relationship would arise. None of the subtests that comprise the NIHTB cognition composites relies on spatial memory or spatial navigation; a skill that is related to right hippocampal volume (Nedelska et al., 2012). Spatial memory and navigation deficits can be an early sign of cognitive dysfunction suggestive of AD (Dudas et al., 2005; Laczó et al., 2018). The current study may be limited in its ability to assess key components of right hippocampal functioning in healthy older adults due to a lack of spatially-based measures that comprise typical cognitive batteries. Although the NIHTB picture sequence memory subtest does have visual and verbal components, this subtest does not assess spatial memory or navigation (Dikmen et al., 2014). This limitation is represented across cognitive batteries and is partly due to a lack of validated spatial navigation assessments for clinical use (Possin, 2010). Considering volumetric asymmetry may not be a sensitive method of understanding the relationship between hippocampal volume and cognition in healthy older adults, the ability to independently assess the contribution of lateral hippocampi to cognitive functioning is perhaps crucial for documenting the nature of the pathological cognitive decline.

### Future Directions and Limitations

While this article focused solely on cognitively intact older adults, there is a need to extend these analyses to include low functioning older adults. More accurate quantification of how lateralized and asymmetrical hippocampal volume contribute to the trajectory of cognitive decline is possible by utilizing a longitudinal method. Additionally, despite known right hippocampal involvement in spatial memory and spatial navigation, and decline of these abilities in AD, these measures are not part of the core functional battery of the NIHTB-CB. Including spatial navigation measures regularly in cognitive batteries will allow us to better quantify changes of right hippocampal volume and function.

While our sample includes a wide range of education (12–21 years), the mean education was about 16 years. In 2015, only 27% of older adults reported attaining a bachelor’s degree or more (U.S. Census Bureau, 2015). Despite controlling for educational attainment in our statistical models, education is a known predictor of cognitive reserve and therefore may have influenced our findings (Farfel et al., 2013). Future studies would benefit from confirming the relationship of hippocampal volume and cognition in a population of older adults that reflects national educational demographics. Additionally, rates of dementia differ between racial and ethnic groups. Non-white individuals develop dementia at a younger age and a higher prevalence (National Research Council, and Committee on Population, 2004; DeCarli et al., 2008). Considering the sample used for this study was predominately non-Hispanic whites, future studies would benefit from exploring racial differences in lateralized hippocampal volume and cognitive functioning.

## Conclusion

The findings from this study contribute to our understanding of the role of lateral and asymmetrical hippocampal volume with multiple domains of cognitive functioning in healthy older adults. This work replicates the relationship between total hippocampal volume and episodic memory. Furthermore, these results show that the strongest relationship of predominately verbal-based episodic memory is with the left hippocampus; suggesting laterality of cognitive functioning in the context of healthy aging. Interestingly, we observed that the asymmetry of the hippocampi appears to not yet be related to cognitive functioning. However, that is not to say asymmetry may not be related as the trajectory of cognitive decline progresses. These findings help to better understand the role of the hippocampus in specific domains of cognitive functioning in healthy older adults and therefore may serve as a foundation to better understand the continuum from healthy to pathological aging.

## Data Availability Statement

The data analyzed in this study is subject to the following licenses/restrictions: data are managed under the data sharing agreement established with NIA and the parent R01 clinical trial Data Safety and Monitoring Board in the context of an ongoing Phase III clinical trial (ACT study, R01AG054077). All trial data will be made publicly available 2 years after completion of the parent clinical trial, per NIA and DSMB agreement. Requests for baseline data can be submitted to the ACT Publication and Presentation (P&P) Committee and will require submission of a data use, authorship, and analytic plan for review by the P&P committee (ajwoods@phhp.ufl.edu). Requests to access these datasets should be directed to ajwoods@ufl.edu.

## Ethics Statement

The studies involving human participants were reviewed and approved by University of Florida Institutional Review Board; University of Arizona Institutional Review Board. The patients/participants provided their written informed consent to participate in this study.

## Author Contributions

CH, AO’S, JK, AA, NE, HH, EB, EV, PB, SD, GA, and AW contributed text to the manuscript. CH, JK, and AO’S contributed to data analysis and processing. All authors contributed to the article and approved the submitted version.

## Conflict of Interest

The authors declare that the research was conducted in the absence of any commercial or financial relationships that could be construed as a potential conflict of interest.

## Footnotes

^1^https://surfer.nmr.mgh.harvard.edu/fswiki/HippocampalSubfieldsAndNucleiOfAmygdala
